# Spatial Confinement Modulated Ru/WO_3_ Heterointerface for Tandem Nitrate-to-Ammonia Conversion in Neutral Electrolytes

**DOI:** 10.3390/ijms27167443

**Published:** 2026-08-20

**Authors:** Zhijiao Ji, Xiaofang Zhang, Wen Gan, Qingzhen Wang, Ming Xu, Luchan Lin, Chufu Li

**Affiliations:** 1 National Institute of Clean-and-Low-Carbon Energy, Beijing 102211, China; 20089955@ceic.com (Z.J.); 17240213@ceic.com (X.Z.); 20090355@ceic.com (W.G.); 20100423@ceic.com (Q.W.); 17240224@ceic.com (M.X.); 2 Shanghai Key Laboratory of Materials Laser Processing and Modification, School of Materials Science and Engineering, Shanghai Jiao Tong University, Shanghai 200240, China

**Keywords:** electrocatalysis, nitrate reduction, laser nano-welding, heterojunction, spatial confinement, series catalysis

## Abstract

To address the challenges of weak NO_3_^−^ adsorption, insufficient active hydrogen supply, and facile desorption of NO_2_^−^ intermediates in neutral electrocatalytic nitrate reduction reaction (NO_3_RR), this study employs laser nano-welding technology to fabricate a Ru/WO_3_ heterojunction, and constructs a Ru/WO_3_/Cu(OH)_2_/FC spatially confined electrode using Cu(OH)_2_ nanorod arrays as the support. Laser welding achieves metallurgical-grade bonding between Ru and WO_3_ while retaining oxygen vacancies in WO_3_. Cu(OH)_2_ promotes NO_3_^−^ adsorption via electrostatic and Lewis acid interactions, and its nanorod array structure confines NO_2_^−^ intermediates. In 0.5 M K_2_SO_4_ + 50 mM KNO_3_ electrolyte, the electrode delivers an ammonia yield rate of 16.1 mg h^−1^ cm^−2^ and a Faradaic efficiency of 75.8% at −0.8 V vs. RHE, outperforming control groups. Potential-dependent electrochemical impedance spectroscopy (EIS) confirms that spatial confinement suppresses NO_2_^−^ accumulation and optimizes interfacial charge transfer kinetics, providing a new strategy for electrode design in neutral NO_3_RR.

## 1. Introduction

Anthropogenic perturbation to the global nitrogen cycle has emerged as one of the most pressing environmental challenges of the 21st century [1]. Excessive nitrate (NO_3_^−^) discharge from agricultural runoff, industrial wastewater, and decentralized sewage not only threatens aquatic ecosystems via eutrophication but also poses direct risks to human health through methemoglobinemia and the potential carcinogenicity of N-nitroso byproducts [2]. Electrocatalytic nitrate reduction to ammonia (NO_3_RR) represents an elegant dual-functional strategy: it enables remediation of nitrate-contaminated water bodies while facilitating green ammonia synthesis using renewable electricity [3]. Among various electrolyte pH windows, neutral media are particularly appealing, as they minimize corrosion, eliminate the need for strong acid/base infrastructure, and are directly compatible with most natural nitrate-containing water bodies [4,5,6]. However, this system faces critical bottlenecks including weak NO_3_^−^ adsorption and insufficient supply of active hydrogen species [7,8].

Tungsten trioxide (WO_3_) and its oxygen-deficient derivative WO_3−x_ have garnered extensive attention as supports for Ru to construct heterointerfaces (Ru/WO_3−x_) featuring a division of labor between “hydrogen reservoirs” and “hydrogenation centers”, owing to their reversible bulk hydrogen intercalation capability and intrinsically low hydrogen evolution reaction (HER) activity. Fu et al. reported mirror-symmetric nanoneedle assemblies composed of c-axis-oriented single-crystalline WO_3_ nanoneedles, and designed an oxygen-deficient MSN-WO_3−x_ to anchor ultrafine Ru nanoclusters for neutral NO_3_RR [9], achieving an ammonia yield rate of 12.38 mg h^−1^ cm^−2^ at −0.6 V vs. RHE. In situ surface-enhanced Raman spectroscopy (SERS) combined with theoretical calculations demonstrated that WO_3−x_ undergoes electrochemical-induced bulk hydrogen intercalation prior to NO_3_RR, transforming into a dynamic active phase of Ru/MSN-H_y_WO_3−x_. The “hydrogen pump” effect locally enriches interfacial *H concentration, thereby accelerating the NO_2_^−^ → NH_3_ hydrogenation step. Zhu et al. used (NH_4_)_x_WO_3_ nanorods hydrothermally grown on Ti felt as precursors, adsorbed Ru^3+^ followed by calcination in a reducing atmosphere to obtain Ru/WO_3−x_ catalysts [10]. Deuterium isotope labeling and differential electrochemical mass spectrometry successfully captured key deuterated intermediates, confirming that protons intercalated into WO_3−x_ migrate to adjacent Ru sites under electric field driving, forming a “reverse hydrogen spillover” channel that significantly accelerates NO_3_RR hydrogenation kinetics. The above two studies collectively demonstrate that the core advantage of Ru/WO_3−x_ heterointerfaces lies in the spatial decoupling of “proton storage sites (WO_3−x_)” and “hydrogenation sites (Ru)”. However, existing Ru/WO_3−x_ catalysts are mostly constructed via hydrothermal coupling with impregnation–calcination methods, where the weak bonding between Ru and WO_3−x_ leads to easy detachment under high-current gas bubble evolution. Meanwhile, the anchoring capacity of active sites for NO_2_^−^ intermediates is weakened in neutral systems, desorbed NO_2_^−^ rapidly enters the electrolyte, causing NO_2_^−^ leakage, which ultimately reduces ammonia yield rate and Faradaic efficiency (FE).

Based on this, this work employs laser nano-welding technology to successfully prepare Ru/WO_3_ heterojunction catalysts. Under pulsed laser irradiation, metal/oxide nanoparticles absorb photons to generate localized photothermal effects [11,12,13,14], realizing melting and recrystallization of WO_3_ and Ru at the nanoscale. The rapid cooling process retains abundant oxygen vacancies in WO_3_ [15], ultimately establishing a metallurgical-grade welding interface. Meanwhile, to improve catalytic efficiency and mitigate *NO_2_^−^ leakage, this work uses Cu(OH)_2_ nanorod arrays as supports to construct a Ru/WO_3_/Cu(OH)_2_ spatially confined electrode structure. The hydrophilic Cu(OH)_2_ nanorod arrays provide high specific surface area [16], promote NO_3_^−^ adsorption via electrostatic and Lewis acid interactions, and the Cu^+^/Cu^2+^ redox pair facilitates the dehydrogenation step of NO_3_^−^ → NO_2_^−^ [17], while Ru is responsible for the subsequent catalytic hydrogenation of NO_2_^−^. WO_3_, which is rich in oxygen vacancies, acts as a “proton reservoir”, providing sufficient protons for the entire reaction process. More importantly, the highly porous structure formed by the Ru/WO_3_ and Cu(OH)_2_ nanorod arrays effectively increases the probability of collisions between the NO_3_RR intermediate and active sites, ultimately leading to a significant improvement in ammonia yield rate and FE. Electrochemical tests indicate that this electrode structure indeed exhibits excellent NO_3_RR performance, reaching an ammonia yield rate of 16.1 mg h^−1^ cm^−2^ and a FE of 75.8% at −0.8 V vs. RHE in neutral electrolyte containing 50 mM NO_3_^−^.

## 2. Results

**Figure 1:**
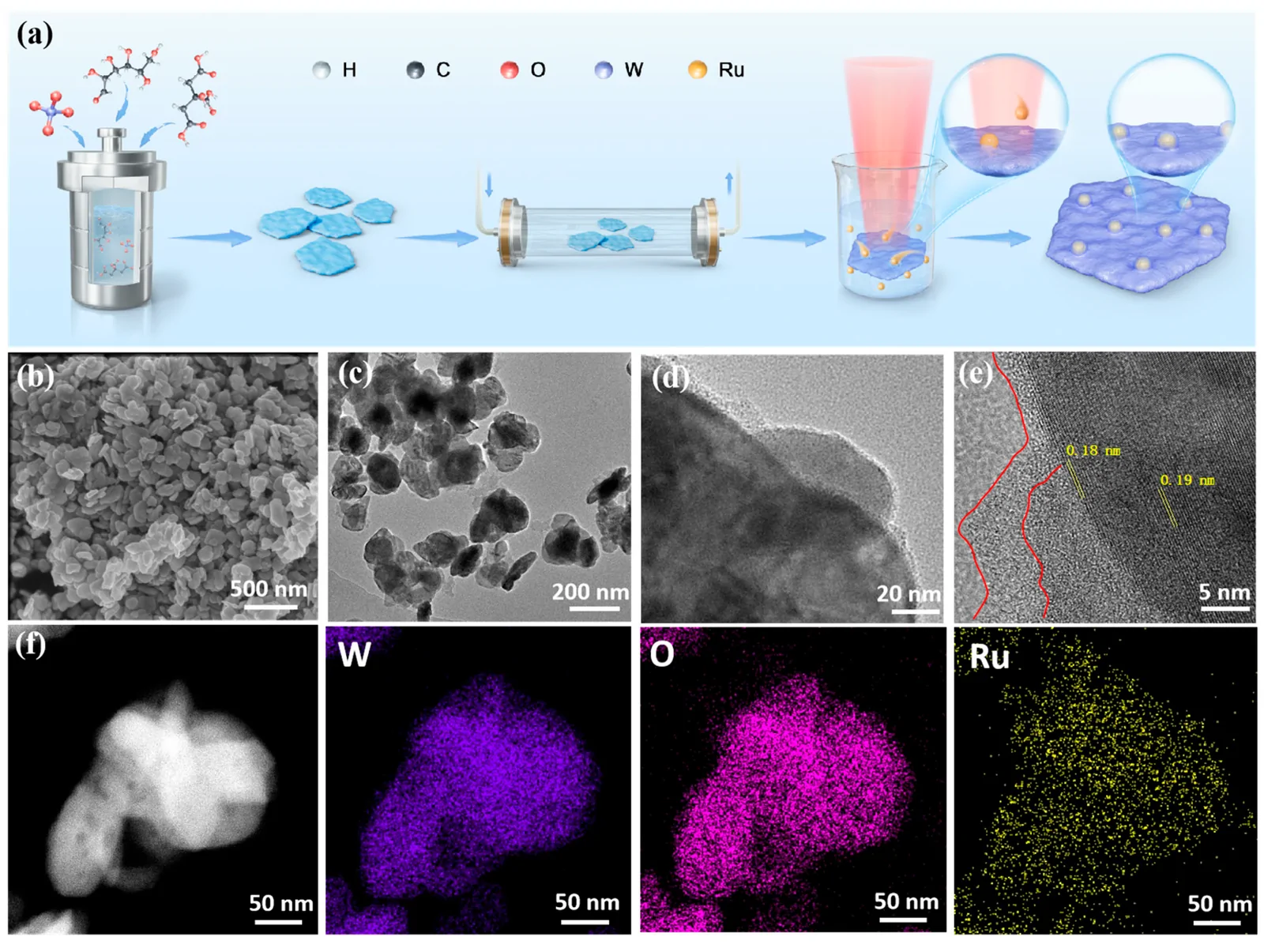
(**a**) Schematic diagram of the Ru/WO_3_ preparation process. (**b**) SEM image, (**c**–**e**) TEM images of Ru/WO_3_. (**f**) EDS element mapping of Ru/WO_3_.

Figure 1 shows the schematic diagram of preparing Ru/WO_3_ heterojunction catalysts via laser nano-welding technology. During laser irradiation, metal nanoparticles migrate toward oxide nanoparticles due to dynamic thermal and force effects, and oxides may be ablated and split to form a plasma plume in the solution. With dynamic movement within local regions, the ablated oxides redeposit onto the surface of metal nanoparticles, forming a thin oxide encapsulation layer [18,19,20]. Figure 1b,c show that the as-prepared Ru/WO_3_ exhibits a nanosheet morphology, consistent with that of pristine WO_3_ (Figure S1), indicating that ultrafast laser irradiation does not alter the morphology of the WO_3_ support. Figure 1d,e reveal that the surface of WO_3_ nanosheets is wrapped with ultrafine Ru nanoparticles, and an amorphous layer forms at the edges. The lattice spacings of 1.8 Å and 1.9 Å correspond to the (400) crystal plane of WO_3_. Elemental mapping results confirm the uniform distribution of Ru nanoparticles on the surface of WO_3_ nanosheets, and the ICP-MS result show that the mass fraction of Ru is 1.65wt%.

**Figure 2:**
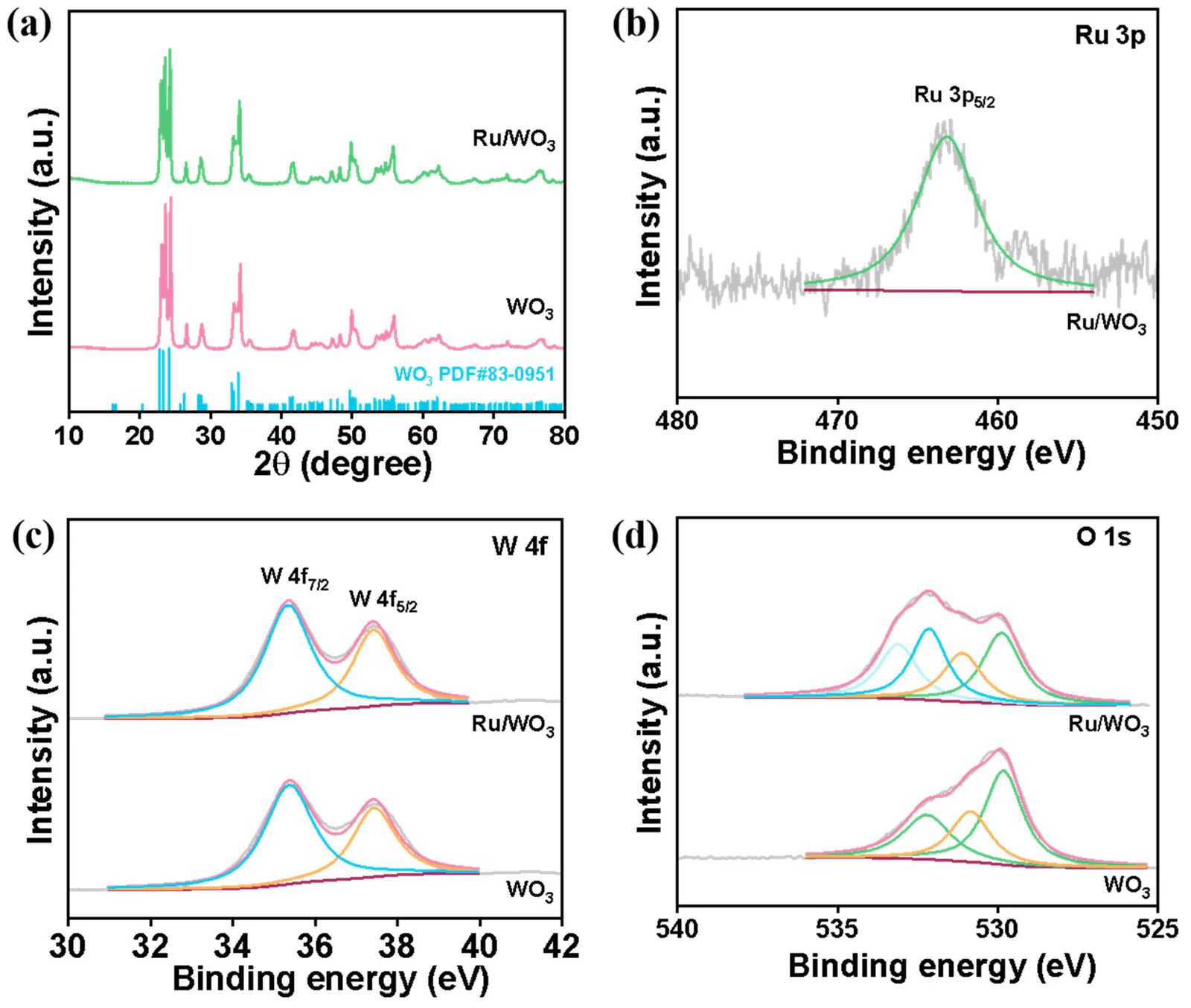
(**a**) XRD patterns of WO_3_ and Ru/WO_3_. XPS spectra of (**b**) Ru 3p, (**c**) W 4f, (**d**) O 1s for WO_3_ and Ru/WO_3_.

X-ray diffraction (XRD) patterns (Figure 2a and Figure S2) and Raman spectra (Figure S3) demonstrate that ultrafast laser irradiation does not affect the crystal structure of WO_3_, and Ru is successfully loaded on the WO_3_ surface. The peak located at 44.1° corresponds to the (101) crystal plane of Ru nanoparticles (PDF#65-7645) (Figure S2). XPS is used to study elemental valence states and electronic structure. As shown in Figure 2b, in the Ru 3p_3/2_ XPS spectrum of Ru/WO_3_, the peak at 463.2 eV is assigned to Ru^0^ [21]. In the W 4f XPS spectrum of Ru/WO_3_ (Figure 2c), the peaks at 35.4 eV and 37.4 eV are attributed to W^6+^ 4f_7/2_ and W^6+^ 4f_5/2_, respectively, showing no obvious shift compared with those of pristine WO_3_ [22]. In the O 1s XPS spectrum of Ru/WO_3_ (Figure 2d), the peaks at 529.9 eV, 531.1 eV, 532.2 eV, and 533.1 eV are assigned to lattice oxygen, oxygen vacancies, chemisorbed H_2_O, and physically adsorbed H_2_O, respectively [23,24,25]. Compared with pristine WO_3_, the characteristic peak of chemisorbed H_2_O in Ru/WO_3_ is significantly enhanced; one of the reasons for this phenomenon is the greater abundance of oxygen vacancies in Ru/WO_3_. These highly active defect sites readily capture water molecules in air and convert them into chemisorbed H_2_O or surface hydroxyl species, directly contributing to the signal intensity in this binding energy range. Electron paramagnetic resonance (EPR) spectroscopy was employed to investigate the defect states of the catalysts. As depicted in Figure S4, both samples exhibit a characteristic single symmetric peak centered at g ≈ 2.003, which is a typical fingerprint for oxygen vacancies (V_O_) in tungsten oxide-based materials [26]. Notably, the sample incorporating Ru nanoparticles displays a significantly enhanced signal intensity compared to the pristine support. The abundant oxygen vacancies in the Ru/WO_3_ heterostructure not only serve as active sites for water dissociation to generate protons but also facilitate the reverse hydrogen spillover process, thereby accelerating the hydrogenation kinetics of nitrate intermediates during the electrocatalytic reduction reaction [27]. In addition, the introduction of metallic Ru nanoparticles provides additional hydrophilic adsorption sites, and their strong chemisorption of water molecules further elevates the relative proportion of this peak. Furthermore, charge redistribution at the Ru-WO_3_ interface enhances the Lewis acidity of W sites, improving the overall water capture and retention capacity of the support, leading to a significant increase in the characteristic peak of physically adsorbed H_2_O [28].

Electrocatalytic NO_3_RR performance evaluation was conducted in an H-type cell with a three-electrode configuration. Since Ru is an essential active site in NO_3_RR, it is necessary to optimize its loading. Therefore, we first drop-coated Ru/WO_3_ samples with Ru loadings of 0.80, 1.65 and 3.33 wt% onto carbon paper (CP) to prepare Ru/WO_3_/CP working electrodes, and evaluated their ammonia yield rate and FE. As shown in Figure S5, the Ru loading of 1.65 wt% exhibited the optimal ammonia yield rate and FE. To enhance the spatial confinement effect of the working electrode for nitrate, Ru/WO_3_ (1.65 wt%) was coated on the surface of electrochemically treated copper foam (Cu(OH)_2_/FC) to obtain the Ru/WO_3_/Cu(OH)_2_/FC electrode. Scanning electron microscopy (SEM) images show that uniform Cu(OH)_2_ nanorod arrays grow on the surface of copper foam after electrochemical treatment (Figure S6), and XRD results confirm the composition as Cu(OH)_2_ (Figure S7). Figure S8 presents the SEM image of the Ru/WO_3_/Cu(OH)_2_/FC electrode, showing that a large number of pores are constructed on the electrode surface supported by Cu(OH)_2_ nanorod arrays, which help increase the residence time of NO_3_^−^ and its intermediates on the electrode surface, thereby improving the NO_3_^−^ → NH_3_ conversion efficiency. As controls, Ru/WO_3_ was also coated on hydrophobic CP and untreated copper foam (FC) to fabricate Ru/WO_3_/CP and Ru/WO_3_/FC electrodes, and SEM images of CP and FC are shown in Figures S9 and S10, respectively.

**Figure 3:**
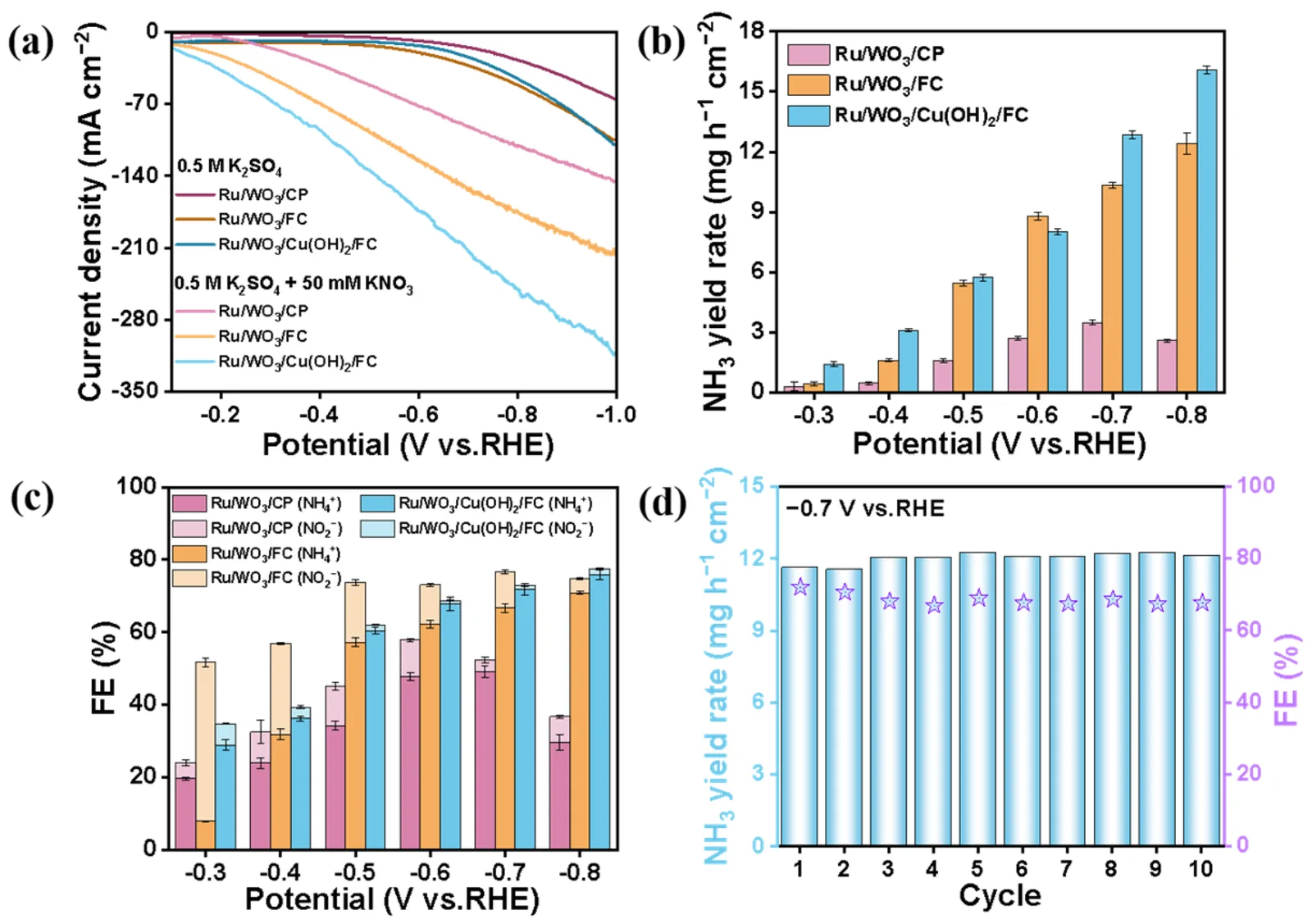
(**a**) LSV curves of Ru/WO_3_/CP, Ru/WO_3_/FC and Ru/WO_3_/Cu(OH)_2_/FC in 0.5 M K_2_SO_4_ and 0.5 M K_2_SO_4_ + 50 mM KNO_3_ electrolyte at the scan rate of 5 mV s^−1^. (**b**) NH_3_ yield rate and (**c**) FE of Ru/WO_3_/CP, Ru/WO_3_/FC and Ru/WO_3_/Cu(OH)_2_/FC in 0.5 M K_2_SO_4_ + 50 mM KNO_3_ electrolyte at different potential. (**d**) The cycling stability of Ru/WO_3_/Cu(OH)_2_/FC in 0.5 M K_2_SO_4_ + 50 mM KNO_3_ electrolyte at −0.7 V vs. RHE.

Linear sweep voltammetry (LSV) curves in Figure 3a shows that the current density increases significantly after adding 50 mM KNO_3_ to 0.5 M K_2_SO_4_ electrolyte, indicating high selectivity of the working electrodes toward NO_3_RR. Moreover, the current density of Ru/WO_3_/Cu(OH)_2_/FC is markedly higher than those of the two control groups. Figure 3b shows that the ammonia yield rate increases with decreasing applied potential. At −0.8 V vs. RHE, the ammonia yield rate of Ru/WO_3_/Cu(OH)_2_/FC reaches 16.1 mg h^−1^ cm^−2^, which is significantly higher than those of Ru/WO_3_/CP (2.6 mg h^−1^ cm^−2^) and Ru/WO_3_/FC (12.4 mg h^−1^ cm^−2^), ranking among the top performers for neutral electrolyte-based NO_3_RR systems.

Figure 3c presents the FE toward ammonia and nitrite for the three electrodes at different potentials in 0.5 M K_2_SO_4_ + 50 mM KNO_3_ electrolyte. The results show that the FE for NO_3_^−^ → NH_3_ over Ru/WO_3_/CP is significantly higher than that for NO_3_^−^ → NO_2_^−^, indicating strong anchoring capacity of Ru/WO_3_ for NO_3_^−^ and its intermediates, so NO_2_^−^ is not prone to desorption into the electrolyte. In contrast, for Ru/WO_3_/FC, the FE for NO_3_^−^ → NO_2_^−^ is significantly higher than that for Ru/WO_3_/CP at low potentials, suggesting that copper foam can accelerate the reduction of NO_3_^−^ to NO_2_^−^, but its adsorption and catalytic reduction capabilities toward NO_2_^−^ are weak. Due to the smooth electrode surface lacking spatial confinement, a large amount of nitrite enters the electrolyte, and this phenomenon is alleviated with continuously decreasing potential. For Ru/WO_3_/Cu(OH)_2_/FC, not only the highest FE toward ammonia is achieved, but the FE for NO_3_^−^ → NO_2_^−^ is also significantly reduced. This indicates that after Cu(OH)_2_/FC efficiently catalyzes NO_3_^−^ reduction to NO_2_^−^, partially desorbed NO_2_^−^ encounters strong spatial confinement by Ru/WO_3_ during diffusion into the electrolyte, which significantly increases the collision probability between NO_2_^−^ and active sites in Ru/WO_3_, enabling re-adsorption of NO_2_^−^ and subsequent stepwise protonation to NH_3_. More importantly, given that no byproducts such as N_2_ and N_2_H_4_ are formed, and that the electrolytic material shows no significant changes in composition or structure after the catalytic reaction, the FE of the hydrogen evolution reaction (HER) can be calculated indirectly based on the FEs of NH_3_ and NO_2_^−^, i.e., FE_H2_ = 1 − FE_NH3_ − FE_NO2_^−^. The results show that the FE of the Ru/WO_3_/Cu(OH)_2_/FC electrode for the HER is even slightly higher than that of the Ru/WO_3_/FC electrode at −0.3 ~ −0.7 V vs. RHE, indicating that the performance gains resulting from the spatial confinement effect created by the Ru/WO_3_ and Cu(OH)_2_/FC structures also extend to the HER.

To further demonstrate that the product NH_3_ originates from the reduction of NO_3_^−^ in the electrolyte, rather than from other nitrogen-containing impurities in the system, we measured the ammonia yield rate of the optimized Ru/WO_3_/Cu(OH)_2_/FC electrode at different potentials in an Ar-saturated 0.5 M K_2_SO_4_ electrolyte. As shown in Figure S11, in the potential range of −0.3 V to −0.8 V vs. RHE, the maximum ammonia yield rate was only 7.4 μg h^−1^ cm^−2^, with an FE below 0.1%. In contrast, the ammonia yield rate of this electrode in a 0.5 M K_2_SO_4_ + 50 mM KNO_3_ electrolyte reached as high as 16.1 mg h^−1^ cm^−2^; the ammonia yield rate increased by approximately 2176-fold. Therefore, the interference caused by impurities in the system can be considered negligible. Figure S12 shows the optimization of Ru/WO_3_ loading on Cu(OH)_2_/FC. The results shows that when the loading exceeded 1 mg cm^−2^, neither the ammonia yield rate nor the FE showed any significant improvement. Table S1 shows that the composite electrode material we designed based on the spatial confinement effect exhibits high ammonia synthesis efficiency in a neutral electrolyte containing low concentrations of NO_3_^−^.

Cyclic stability tests demonstrate that the developed Ru/WO_3_/Cu(OH)_2_/FC electrode maintains high catalytic activity and FE even after 10 consecutive cycles (Figure 3d). Figure S13 shows the long-term chronoamperometry tests of the Ru/WO_3_/Cu(OH)_2_/FC electrode; due to the rapid consumption rate of NO_3_^−^ in the electrolyte, the current density gradually decreases as the electrolysis time increases. After replacing the electrolyte with a fresh one, the current density returns to its initial value, indicating that the Ru/WO_3_/Cu(OH)_2_/FC electrode exhibits good stability and that the decline in current density is attributable to the decrease in the NO_3_^−^ concentration in the electrolyte. However, after 10 h of continuous electrolysis, the rate of current density decay accelerated significantly, indicating that the catalyst may have begun to degrade.

Thus, we conducted SEM and XRD analyses of Ru/WO_3_/Cu(OH)_2_/FC electrode after a 10-cycle stability test and a long-term chronoamperometry test, respectively, to investigate changes in its morphology and composition. As shown in Figure S14, the morphology of the working electrode remained essentially unchanged, with Ru/WO_3_ nanosheets attached to the Cu(OH)_2_ nanorod array. EDS elemental mapping indicated that Ru was still uniformly distributed. We analyzed the electrolyte from the 2 h electrolysis experiment conducted during the long-term chronoamperometry testing using ICP-MS to determine whether Ru nanoparticles had dissolved. The results showed virtually no Ru-related signals, indicating that Ru nanoparticles can exist stably in this system. In addition to the characteristic elements of the working electrode, we also detected distinct F, S, and K signals in the EDS element mapping, which primarily originated from the Nafion binder used in the preparation of the catalyst slurry and the electrolytes that have not been thoroughly rinsed away. The XRD data shows that the crystal structures of Cu(OH)_2_ and Ru/WO_3_ remained intact (Figure S15). However, after long-term chronoamperometry test, things are different. SEM results show that there was no significant change in the surface morphology of the electrodes (Figure S16), but XRD results showed that the characteristic diffraction peaks of Cu(OH)_2_ disappeared, and the crystal structure of WO_3_ also underwent significant changes (Figure S17). This indicates that the structure of the electrode underwent significant changes under prolonged negative potential and in a high-concentration K_2_SO_4_ electrolyte, leading to rapid performance degradation after 10 h.

**Figure 4:**
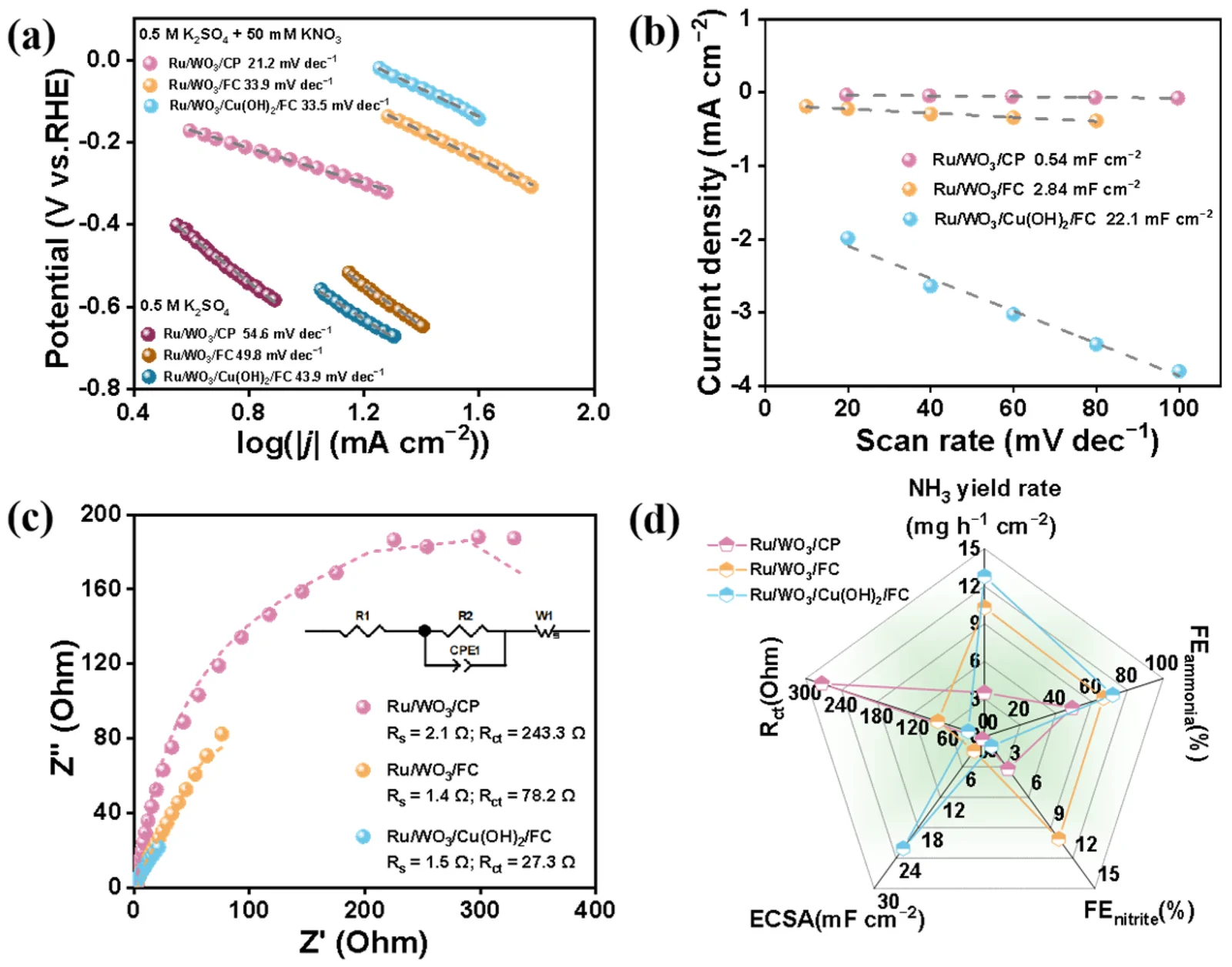
(**a**) Tafel slope of Ru/WO_3_/CP, Ru/WO_3_/FC and Ru/WO_3_/Cu(OH)_2_/FC in 0.5 M K_2_SO_4_ and 0.5 M K_2_SO_4_ + 50 mM KNO_3_ electrolyte. (**b**) C_dl_ of Ru/WO_3_/CP, Ru/WO_3_/FC and Ru/WO_3_/Cu(OH)_2_/FC in 0.5 M K_2_SO_4_ electrolyte. (**c**) EIS curves of Ru/WO_3_/CP, Ru/WO_3_/FC and Ru/WO_3_/Cu(OH)_2_/FC in 0.5 M K_2_SO_4_ + 50 mM KNO_3_ electrolyte at OCP. (**d**) Statistical chart of the electrochemical properties of three types of working electrodes.

Tafel slopes were used to investigate the reaction kinetics of Ru/WO_3_/CP, Ru/WO_3_/FC, and Ru/WO_3_/Cu(OH)_2_/FC in 0.5 M K_2_SO_4_ and 0.5 M K_2_SO_4_ + 50 mM KNO_3_ electrolytes (Figure 4a). The results show that Tafel slopes decrease universally after adding KNO_3_. This is because NO_3_^−^ is selectively adsorbed at the active sites, thereby inhibiting the adsorption of H_2_O and significantly accelerating the reaction kinetics. Surprisingly, Ru/WO_3_/CP exhibits the smallest Tafel slope in KNO_3_-containing electrolyte, indicating fast intrinsic NO_3_RR kinetics of Ru/WO_3_, the poor overall performance is attributed to an insufficient number of active sites and their inadequate ability to stabilize reaction intermediates. Electrochemical active surface area (ECSA) results corroborate this hypothesis well, which is obtained using the double-layer capacitance (C_dl_) method. Figure S18 shows the cyclic voltammetry curves of different electrodes at various scan rates. As shown in Figure 4b, the C_dl_ of Ru/WO_3_/Cu(OH)_2_/FC is 41 times and 8 times that of Ru/WO_3_/CP and Ru/WO_3_/FC, respectively. Based on the equation ECSA = C_dl_/C_s_, the ECSA for Ru/WO_3_/CP, Ru/WO_3_/FC, and Ru/WO_3_/Cu(OH)_2_/FC were calculated to be 13.5, 71.0, and 552.5, respectively, where C_s_ is the capacitance per unit active surface area, typically taken as 40 μF cm^−2^. Figure S19 shows the LSV curves of different working electrodes in a 0.5 M K_2_SO_4_ + 50 mM KNO_3_ electrolyte, normalized using ECSA. The results indicate that the Ru/WO_3_/CP electrode, which had the lowest apparent current density, exhibited the highest intrinsic current density. These results indicate that the Ru/WO_3_ electrode possesses high intrinsic NO_3_RR activity, which is consistent with the Tafel slope results. However, it lacks sufficient active sites and the ability to stabilize the NO_3_RR intermediate. Upon loading it onto a Cu(OH)_2_ nanorod array, it not only increases the ECSA of the working electrode, but also creates a spatial confinement effect, ultimately leading to high synthetic ammonia performance.

Figure 4c shows the EIS spectra of different working electrodes at open circuit potential (OCP). Fitting results indicate that the solution resistance (R_s_) of all systems is low, so no additional iR compensation was performed on current densities. The charge transfer resistance (R_ct_) of Ru/WO_3_/Cu(OH)_2_/FC is only 27.6 Ω, significantly lower than those of Ru/WO_3_/CP (243.3 Ω) and Ru/WO_3_/FC (78.2 Ω), indicating easier interfacial charge transfer in the constructed hierarchical electrode. Figure 4d provides a more intuitive illustration of the electrochemical data for different electrodes. The results show that the improvement in ammonia synthesis performance is primarily attributable to a larger ECSA and a lower R_ct_.

**Figure 5:**
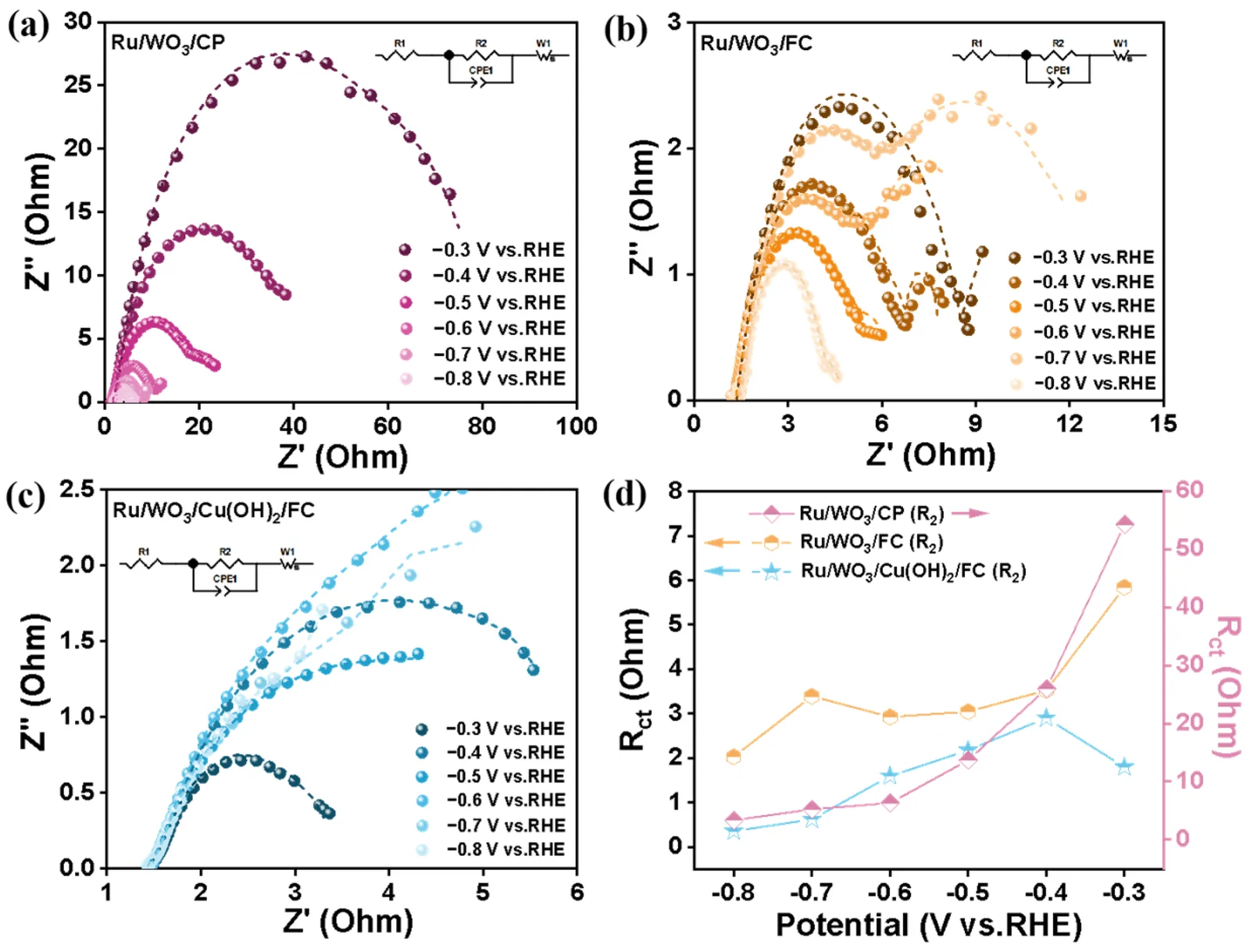
Variable potential EIS curves of (**a**) Ru/WO_3_/CP, (**b**) Ru/WO_3_/FC, (**c**) Ru/WO_3_/Cu(OH)_2_/FC. (**d**) R_ct_ of different electrodes at different potentials.

To further investigate the variation of R_ct_ on different working electrodes during NO_3_RR, potential-dependent EIS measurements were carried out. Figure 5a–c show the EIS spectra of Ru/WO_3_/CP, Ru/WO_3_/FC, and Ru/WO_3_/Cu(OH)_2_/FC in the potential range of −0.3 V to −0.8 V vs. RHE, respectively. The fitted R_s_ and R_ct_ are presented in Figure S20 and Figure 5d. For Ru/WO_3_/CP, R_ct_ decreases gradually with decreasing potential, indicating that the applied overpotential directly promotes the charge transfer process and accelerates interfacial electron transfer kinetics. However, an increase in R_ct_ is observed for both Ru/WO_3_/FC and Ru/WO_3_/Cu(OH)_2_/FC, occurring in the potential ranges of −0.6 ~ −0.7 V vs. RHE for the former and −0.3 ~ −0.4 V vs. RHE for the latter. The increase in R_ct_ for Ru/WO_3_/FC arises from the continuous accumulation of the intermediate NO_2_^−^ during sequential potential-dependent EIS tests, which causes abnormal R_ct_ elevation. However, as the potential becomes more negative, strong convection under high overpotential disrupts the accumulated intermediates, leading to a decrease in R_ct_. Notably, no abnormal R_ct_ elevation is observed for Ru/WO_3_/Cu(OH)_2_/FC at high overpotentials, indicating low accumulation of NO_2_^−^ and negligible impact on the working electrode, which is consistent with the NO_3_RR performance results. The slight R_ct_ increase in the low potential region for Ru/WO_3_/Cu(OH)_2_/FC originates from dynamic reorganization of the surface micro-environment: a small amount of adsorbed OH^−^ on the electrode surface is replaced by NO_3_^−^ under applied potential, leading to a marginal increase in R_ct_ before it continuously decreases with further potential lowering.

## 3. Discussion

In this study, a Ru/WO_3_ heterojunction was constructed via laser nano-welding technology, and combined with the spatial confinement effect of Cu(OH)_2_ nanorod arrays to achieve efficient NO_3_RR to ammonia in neutral electrolyte. The metallurgical interface formed by laser welding enhances the bonding strength between Ru and WO_3_, while Ru as the active site of NO_3_RR and oxygen vacancies in WO_3_ serve as “hydrogen reservoirs” to supply active hydrogen. More importantly, the porous micro-environment constructed by Ru/WO_3_ and Cu(OH)_2_/FC addresses the efficiency loss caused by desorption and leakage of NO_2_^−^ in conventional electrodes. Electrochemical tests demonstrate that the as-fabricated electrode delivers an ammonia yield rate of 16.1 mg h^−1^ cm^−2^ with a FE of 75.8% at −0.8 V vs. RHE. Potential-dependent EIS and Tafel analysis confirm that the spatial confinement structure optimizes interfacial charge transfer and reaction pathways. This study provides a novel strategy for the design of electrodes for neutral NO_3_RR.

## 4. Materials and Methods

### 4.1. Chemical

All chemical reagents were purchased and used directly without further purification. Sodium tungstate dihydrate (Na_2_WO_4_⋅2H_2_O, ≥99%), citric acid monohydrate (C_6_H_8_O_7_⋅H_2_O, ≥99%), D-(+)-Glucose (C_6_H_12_O_6_, ≥99%), potassium sulfate (K_2_SO_4_, 99%), sodium hydroxide (NaOH, 97%), sodium hypochlorite solution (NaClO, AR 6~14%), ethanol (CH_3_CH_2_OH, 99.5%), sodium nitroferricyanide dihydrate (C_5_FeN_6_Na_2_O_2_⋅H_2_O, 99%), Ruthenium (III) chloride trihydrate (RuCl_3_⋅3H_2_O, 99%), L-ascorbic acid (99.99%), Polyvinylpyrrolidone (PVP, M_w_ = 55,000) were purchased from Aladdin Chemical Reagent Company (Shanghai, China). Deionized water was used for all solution preparation and washing procedures. N_2_ (99.999%) and Ar (99.999%) were purchased from Date (Dalian) gas Ltd. (Dalian, China).

### 4.2. Characterization

Morphology of the samples was examined by scanning electron microscope (SEM, FEINova NanoSEM450) with an energy dispersive spectrometer (EDS) (Hillsboro, Oregon, USA). The crystalline structure of the samples was determined by X-ray diffraction (XRD) measurement on a Bruker D8 ADVANCE (Karlsruhe, Baden-Württemberg, Germany), using Cu-Kα 40 kV 40 mA and a scan step of 1.2° min^−1^ in the range of 5° to 90° (2θ). X-ray photoelectron spectroscopy (XPS) was performed on a Thermo Scientific ESCALAB 250 Xi spectrometer equipped with a monochromatic Al Kα X-ray source (1486.6 eV) (East Grinstead, West Sussex, UK). Raman spectroscopy was performed on a horiba HR800 spectrometer using 532 nm laser (Longjumeau, Essonne, France).

### 4.3. Fabrication of Ru/WO_3_/Cu(OH)_2_/FC Working Electrode

Preparation of WO_3_ rich in oxygen vacancies. A total of 2 mmol of Na_2_WO_4_⋅2H_2_O was dispersed in 60 mL of water and stirred to make a uniform dispersion, followed by the addition of 3 mmol citric acid monohydrate and 10 mmol glucose; a clarified and transparent solution was obtained by continuous stirring for 30 min. Subsequently, 3 mL 6 M HCl was added drop by drop. After continuous stirring for 1 h, the suspension was transferred to 100 mL Polytetrafluoroethylene (PTFE) reactor liner and put into a stainless steel reactor, screwed tightly, and then placed in a 120 °C blast drying oven for 24 h. After the reaction was finished, the reactor was cooled down naturally, and the product was taken out and washed thoroughly with water and ethanol, and then placed in a vacuum drying oven at 70 °C for 12 h. The catalyst obtained was designated as WO_3_⋅H_2_O.

An appropriate amount of WO_3_⋅H_2_O was placed in a quartz boat and transferred to a tube furnace, where it was pyrolyzed under an 5% H_2_/Ar atmosphere, and increased from room temperature to 550 °C at an elevation rate of 2 °C min^−1^, and after 2 h calcination it was cooled down naturally to obtain WO_3_ rich in oxygen vacancies.

Preparation of Ru/WO_3_. Ru/WO_3_ powder with a nominal Ru loading of 2 wt% was prepared by a wet-chemical adsorption–reduction method. Briefly, WO_3_ powder (1.00 g), RuCl_3_·3H_2_O (0.041 g) and PVP (0.040 g) were dispersed in 40 mL of deionized water under magnetic stirring (300 r/min) at room temperature for 1 h. L-ascorbic acid (0.10 g) was separately dissolved in 10 mL of deionized water and then added to the above suspension. The mixture was heated to 80 °C and stirred for 2 h to reduce Ru^3+^. After the reaction, the solid product was collected by centrifugation at 8000 rpm for 5 min. The precipitate was then redispersed in 40 mL of deionized water and centrifuged again at 8000 r/min for 5 min. This washing process was repeated three times to remove residual ions, PVP and unreacted reductant. Finally, the collected grey–black precipitate was dried at 60 °C for 12 h to obtain Ru/WO_3_ powder.

Fabrication of Ru/WO_3_/Cu(OH)_2_/FC working electrode. First, pre-cut copper foam (1 × 2 cm) was immersed in HCl solution and ultrasonicated for 15 min to remove surface oxides and organic protective layers, followed by sequential ultrasonic cleaning with ethanol and deionized water twice each for 15 min, and finally blown dry with N_2_ for later use. Second, the cleaned copper foam was immersed in 2 M KOH electrolyte, with an immersion area of 1 × 1 cm, and pretreated under chronopotentiometry mode at a current density of −20 mA cm^−2^ for 20 min to grow Cu(OH)_2_ nanorod arrays.

For catalyst ink preparation, 5 mg of catalyst was weighed, followed by the addition of 1928 μL of dispersant (V_isopropanol_:V_water_ = 3:1) and 72 μL of 5 wt% Nafion solution. After ultrasonication for 1 h, 400 μL of the catalyst ink was pipetted evenly onto the surface of the as-prepared Cu(OH)_2_-modified copper foam to obtain the Ru/WO_3_/Cu(OH)_2_/FC electrode, and the loading capacity of Ru/WO_3_ is 1 mg cm^−2^. For comparison, Ru/WO_3_ was also coated on hydrophobic carbon paper (CP) and pristine copper foam (FC) to fabricate Ru/WO_3_/CP and Ru/WO_3_/FC electrodes following the same coating procedure.

### 4.4. Electrochemical Testing

Electrochemical testing was carried out using a Princeton 4000 A electrochemical workstation with an H-type electrolyzer separated by a Nafion 211 membrane. The anode electrolyte is 0.5 M K_2_SO_4_, and the cathode electrolyte is 0.5 M K_2_SO_4_ + 50 mM KNO_3_. Unless otherwise specified, three parallel experiments were conducted for both the ammonia yield rate and FE tests.

A saturated Ag/AgCl electrode and a platinum sheet (1 × 1.5 cm) were used as the reference and counter electrodes, respectively. All potentials were calibrated to the reversible hydrogen electrode (RHE) based on the equation:

E (vs. RHE) = E (vs. Ag/AgCl) + 0.059 × pH + 0.198

### 4.5. Determination and Quantitation of Ammonia Using UV-Vis

The concentration of product NH_3_ was determined using the indophenol blue method. Typically, 2 mL electrolyte was taken and added into 2 mL 1 mol L^−1^ NaOH solution containing 5 wt% salicylic acid and 5 wt% sodium citrate, followed by further addition of 1 mL 0.05 mol L-1 NaClO and 0.2 mL of 1 wt% C_5_FeN_6_Na_2_O. After incubating for 2 h, the absorbance measurement of the above solutions was performed at = 655 nm. The concentration fitting curve (y = 0.47 x + 0.0032, R^2^ = 0.999) consistently showed a good linear relationship between the absorbance value and NH_3_ concentration by independently calibrating three times.

### 4.6. Determination and Quantitation of Nitrite Using UV-Vis

A certain amount of electrolyte was removed from the electrolytic cell and diluted to 5 mL to the detection range. Then, 0.1 mL of nitrite color reagent was added to the aforementioned solution. After 1 h, the absorption spectrum was tested using a UV–vis spectrophotometer, and the absorption intensities at a wavelength of 540 nm were recorded. The nitrite color reagent consisted of 40 g L^−1^ p-aminobenzenesulfonamide, 100 mL L^−1^ phosphoric acid and 2 g L^−1^ N-(1-naphthyl)-ethylenediamine dihydrochloride. The concentration fitting curve (y = 0.875 x + 0.0095, R^2^ = 0.999) consistently showed a good linear relationship between the absorbance value and nitrite concentration by independently calibrating three times.

### 4.7. Faradaic Efficiency

Faradaic efficiency was calculated by the following equation:

$$Faradaic\;efficiency(\%)=\frac{Q_{NH3}}{Q}=\frac{n_{NH3}\times V\times c_{{NH}_{3}}\times F}{Q}\times 100\%$$

where Q represents the applied overall coulomb quantity (C), Q_NH3_ is the coulomb required to produce NH_3_, $n_{NH3}$ is the electron-transfer number (for 1 mol NH_3_, it is 8), V is the volume of the catholyte of the cathode chamber (30 mL), c_NH3_ is the concentration of NH_3_ produced, and F is the Faraday constant (96,485 C mol^−1^).

## Data Availability

The original contributions presented in this study are included in the article/Supplementary Materials. Further inquiries can be directed to the corresponding author(s).

## Supplementary Materials

The following supporting information can be downloaded at https://www.mdpi.com/article/10.3390/ijms27167443/s1. References [29,30,31,32,33,34,35] are cited in Supplementary Materials.

## Abbreviations

The following abbreviations are used in this manuscript:

Ru/WO_3_/CP	Ru/WO_3_ deposited on carbon paper
Ru/WO_3_/FC	Ru/WO_3_ supported on foam copper
Ru/WO_3_/Cu(OH)_2_/FC	Ru/WO_3_ supported on foam copper coated with an array of Cu(OH)_2_ nanorods
FE	Faraday Efficiency
CV	Cyclic Voltammetry
LSV	Linear Sweep Voltammetry
Cdl	Double-Layer Capacitance
ECSA	Electrochemically Active Surface Area
Rs	Ohmic Impedance
Rct	Charge Transfer Impedance
XRD	X-ray Diffraction
SEM	Scanning Electron Microscope
TEM	Transmission Electron Microscope
EDS	Energy Dispersive Spectroscopy
XPS	X-ray Photoelectron Spectroscopy
ICP-MS	Inductively Coupled Plasma Mass Spectrometer
