# Supplementary material for: Spatial Confinement Modulated Ru/WO3 Heterointerface for Tandem Nitrate-to-Ammonia Conversion in Neutral Electrolytes
Source: Int J Mol Sci. 2026 Aug 20;27(16):7443. doi: 10.3390/ijms27167443 (PMC13513373; doi:10.3390/ijms27167443)
Supplement: Supplementary file 1 [file ijms-27-07443-s001.zip › ijms-4465285-supplementary.pdf]

Supporting Information

Spatial Confinement Modulated Ru/WO3 Heterointerface for Tandem Nitrate-to-Ammonia Conversion in Neutral Electrolytes

Zhijiao Ji [1], Xiaofang Zhang [1], Wen Gan [1], Qingzhen Wang [1], Ming Xu [1], Luchan Lin [2,*] and Chufu Li [1,*]

[1] National Institute of Clean-and-Low-Carbon Energy, Beijing 102211, China; 20089955@ceic.com (Z.J.); 17240213@ceic.com (X.Z.); 20090355@ceic.com (W.G.); 20100423@ceic.com (Q.W.); 17240224@ceic.com (M.X.)

[2] Shanghai Key Laboratory of Materials Laser Processing and Modification, School of Materials Science and Engineering, Shanghai Jiao Tong University, Shanghai 200240, China

* Correspondence: luchan.lin@sjtu.edu.cn (L.L.); 17240117@ceic.com (C.L.)

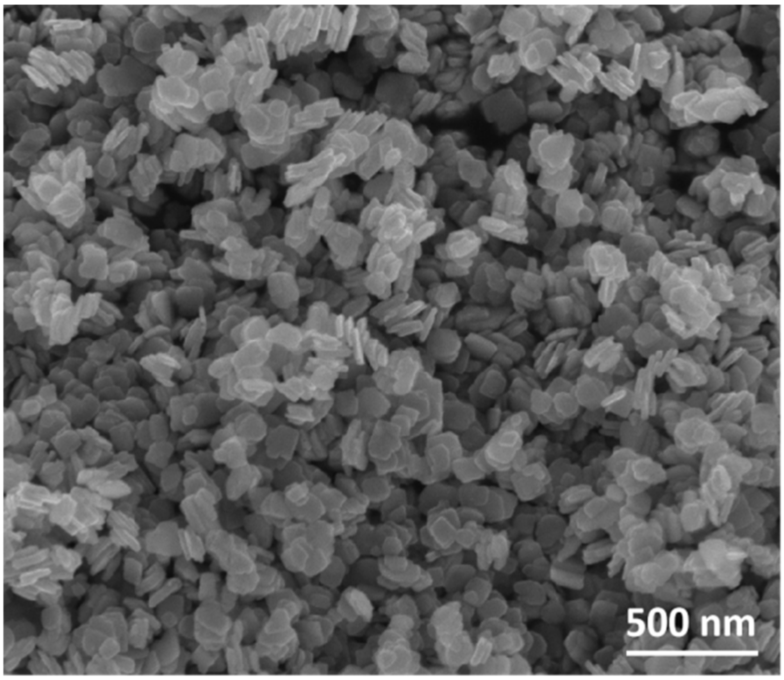

Figure S1. SEM image of WO_3.

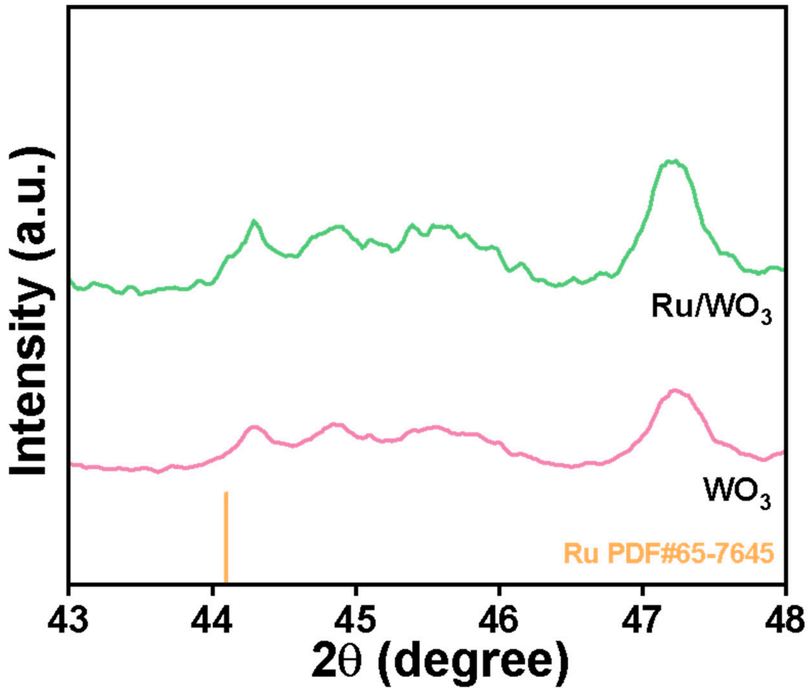

Figure S2. XRD patterns of WO_3 and Ru/WO_3.

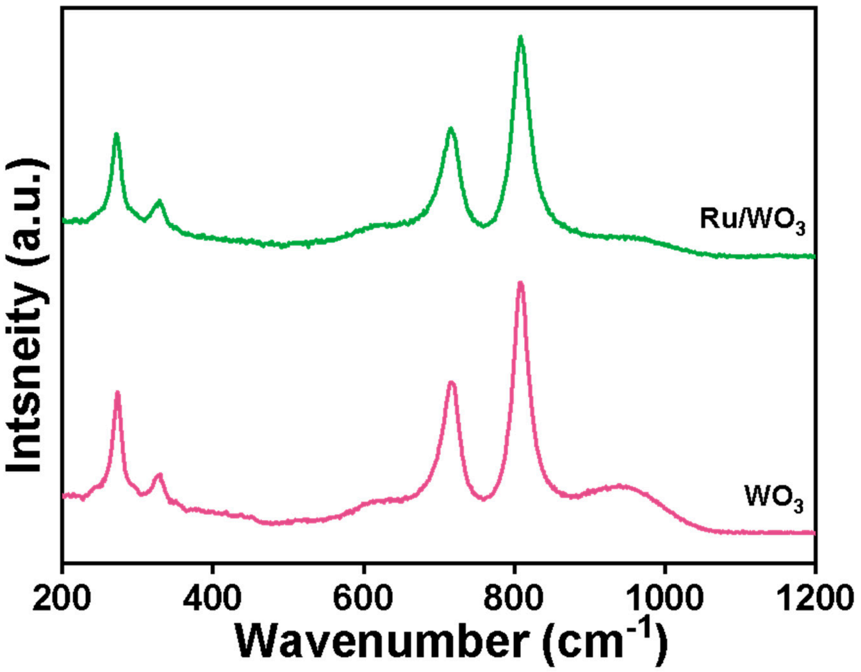

Figure S3. Raman spectra of WO_3 and Ru/WO_3.

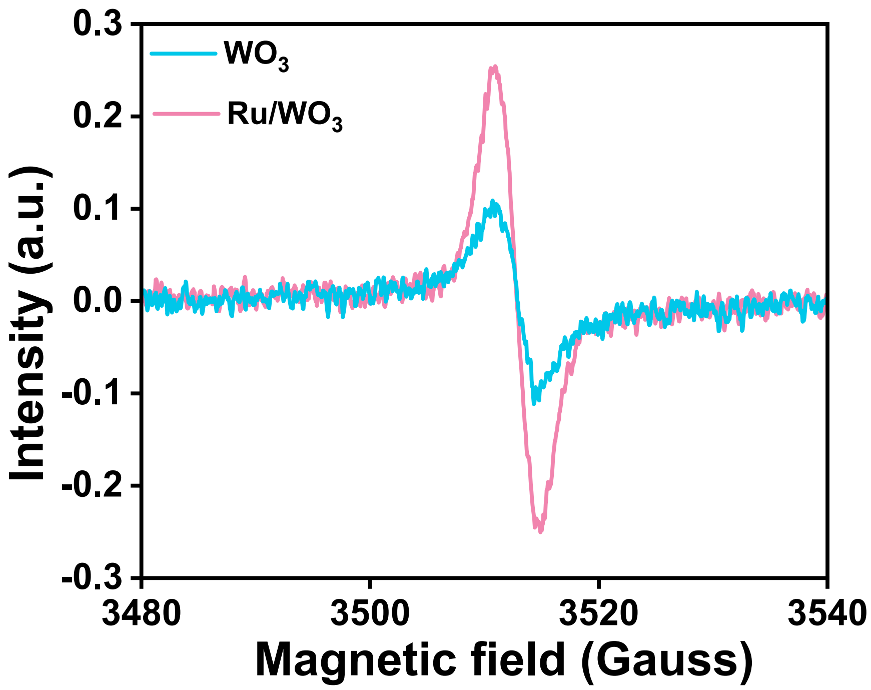

Figure S4. EPR results of WO_3 and Ru/WO_3.

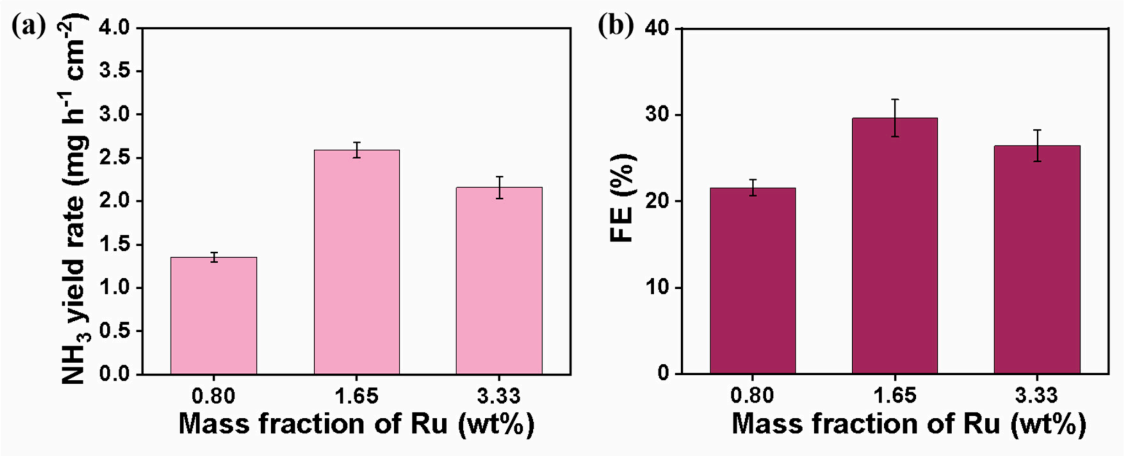

Figure S5. (a) Ammonia yield rate and (b) FE of Ru/WO_3/$Cu(OH)_2$/FC electrodes with different Ru mass fractions at -0.8 V vs.RHE in a 0.5 M K_2SO_4 + 50 mM KNO_3 electrolyte.

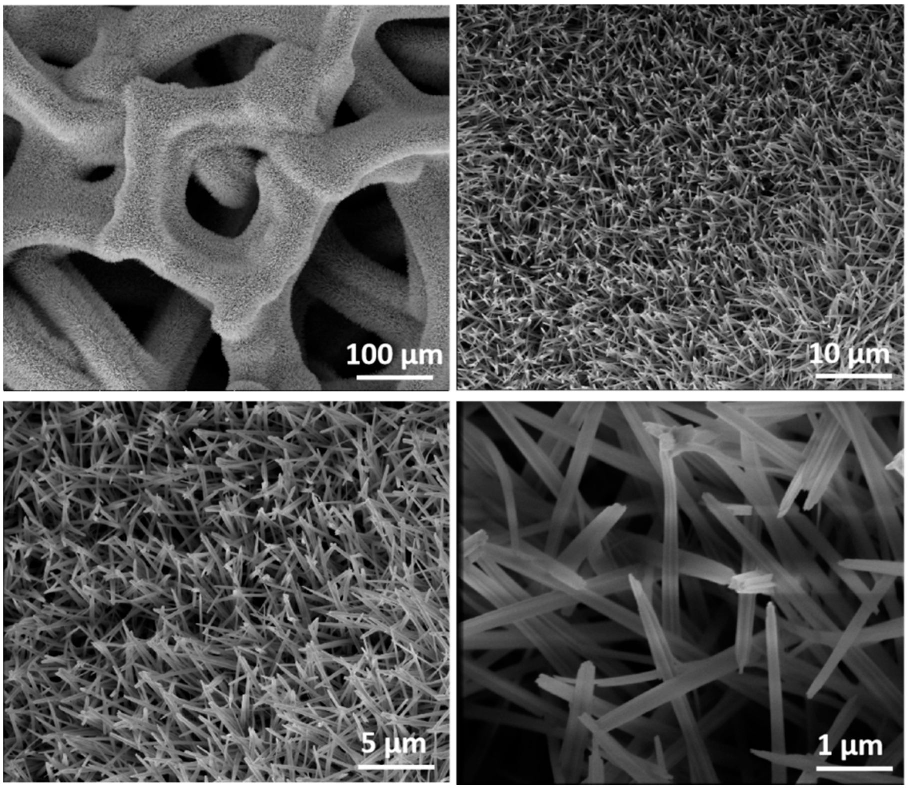

Figure S6. SEM images of $Cu(OH)_2$/FC at different scales.

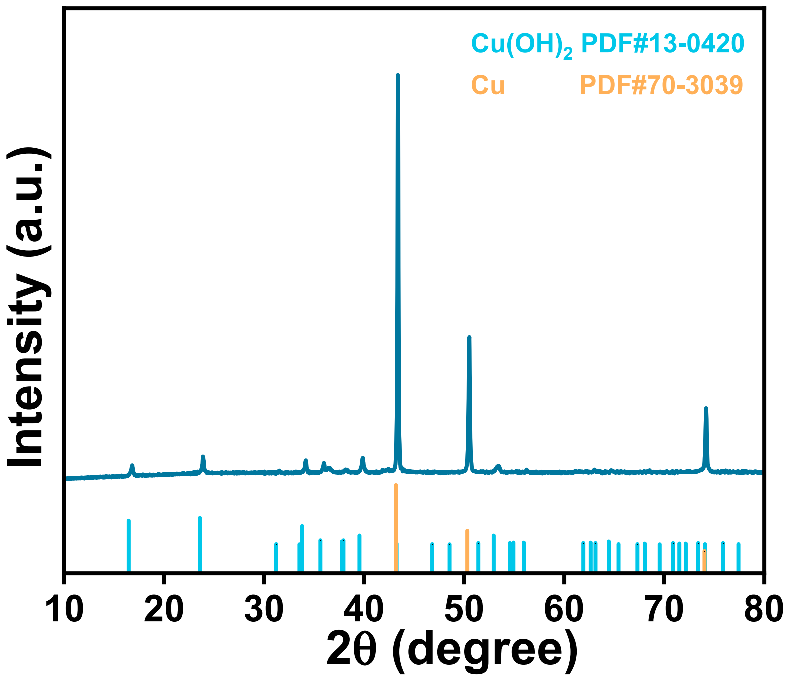

Figure S7. XRD pattern of $Cu(OH)_2$/FC.

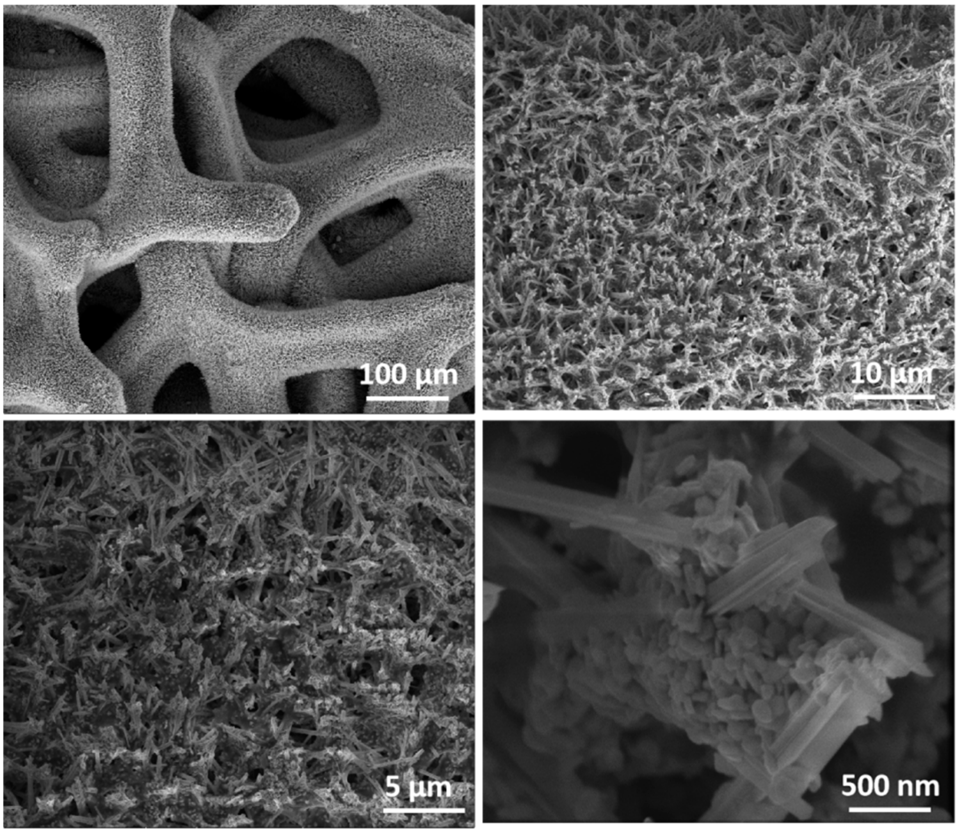

Figure S8. SEM images of Ru/WO_3/$Cu(OH)_2$/FC at different magnification.

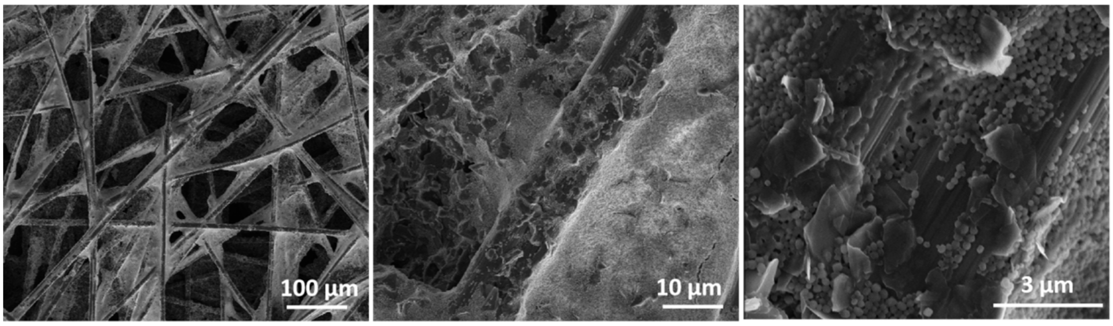

Figure S9. SEM images of hydrophobic carbon paper at different magnification.

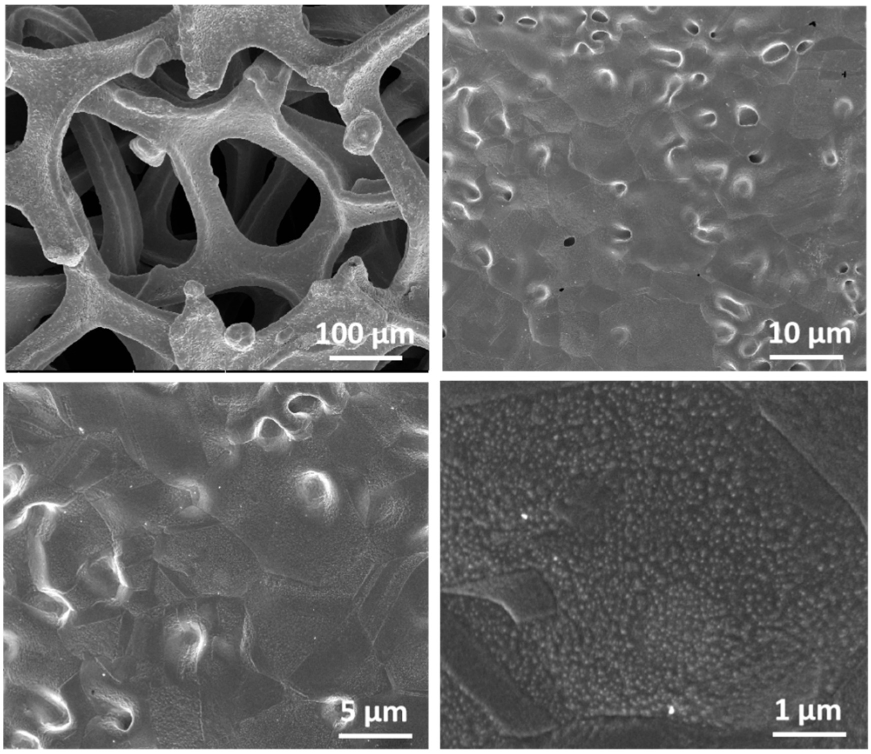

Figure S10. SEM images of copper foam at different magnification.

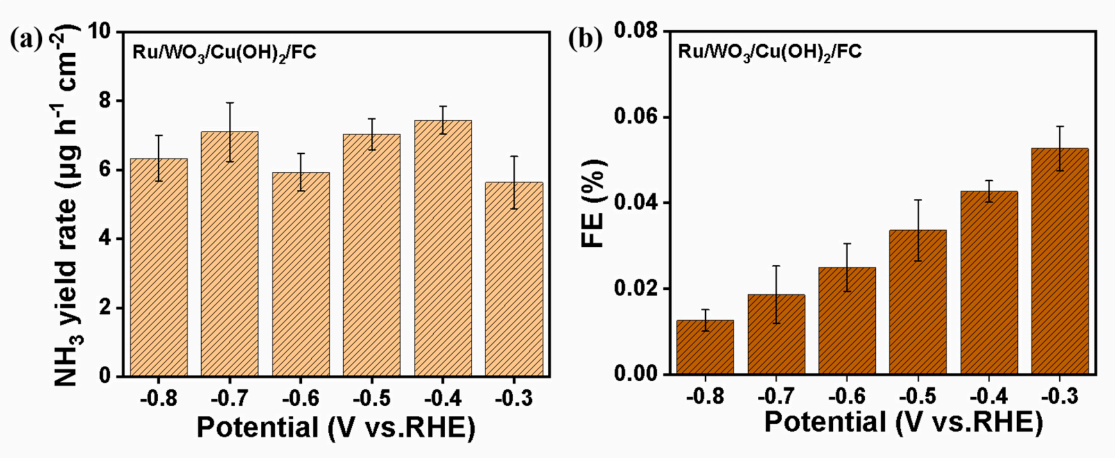

Figure S11. (a) Ammonia yield rate and (b) FE of $Ru/WO_3/Cu(OH)_2$/FC electrodes in Ar-saturated 0.5 M K_2SO_4 electrolyte at different potential.

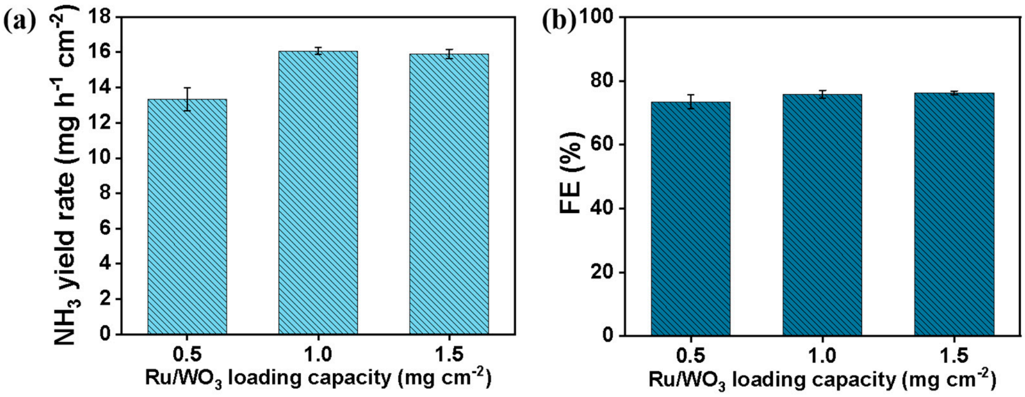

Figure S12. (a) Ammonia yield rate and (b) FE of $Ru/WO_3/Cu(OH)_2$/FC electrodes with different Ru/WO_3 loading capacity at -0.8 V vs.RHE in a 0.5 M K_2SO_4 + 50 mM KNO_3 electrolyte.

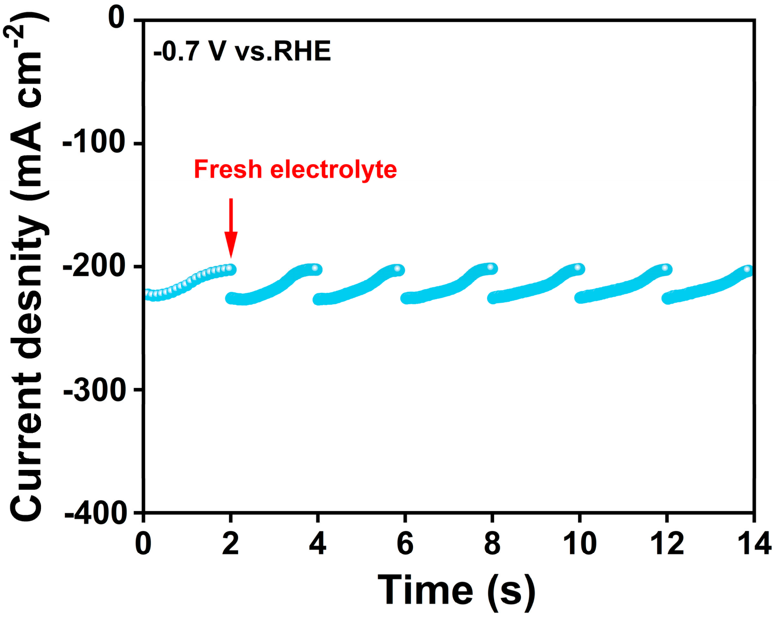

Figure S13. Long-term chronoamperometry curves of Ru/WO_3/$Cu(OH)_2$/FC electrodes electrodes in a 0.5 M K_2SO_4 + 50 mM KNO_3 electrolyte.

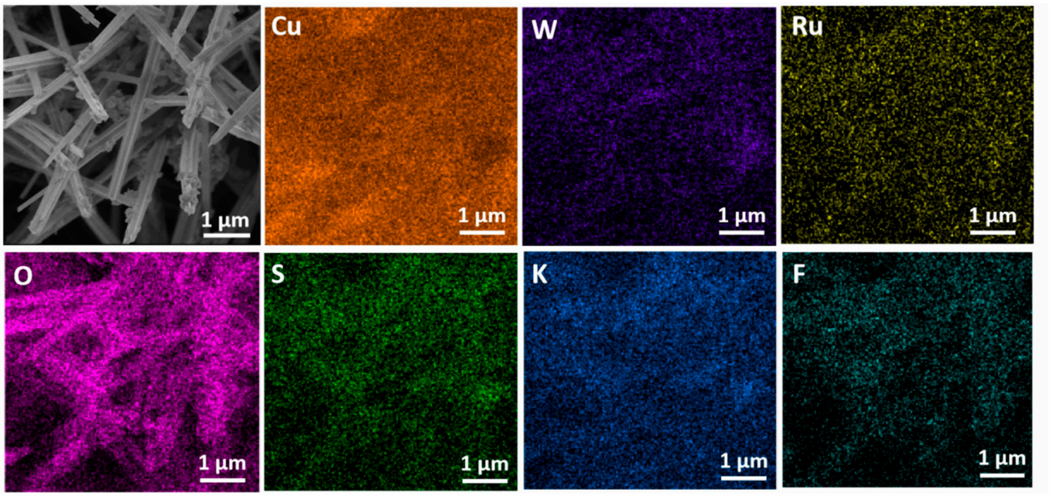

Figure S14. SEM and EDS elemental mapping of the Ru/WO_3/$Cu(OH)_2$/FC electrode after 10 cycle tests.

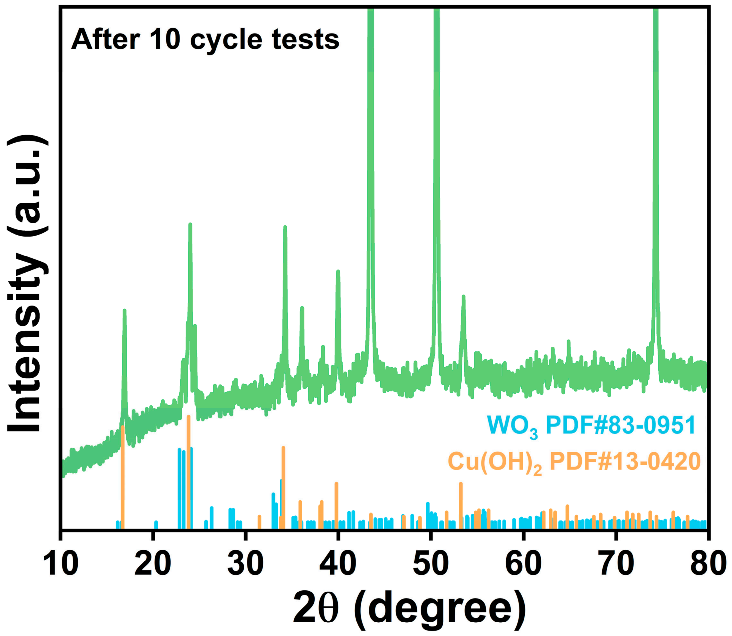

Figure S15. XRD pattern of the Ru/WO_3/$Cu(OH)_2$/FC electrode after 10 cycle.

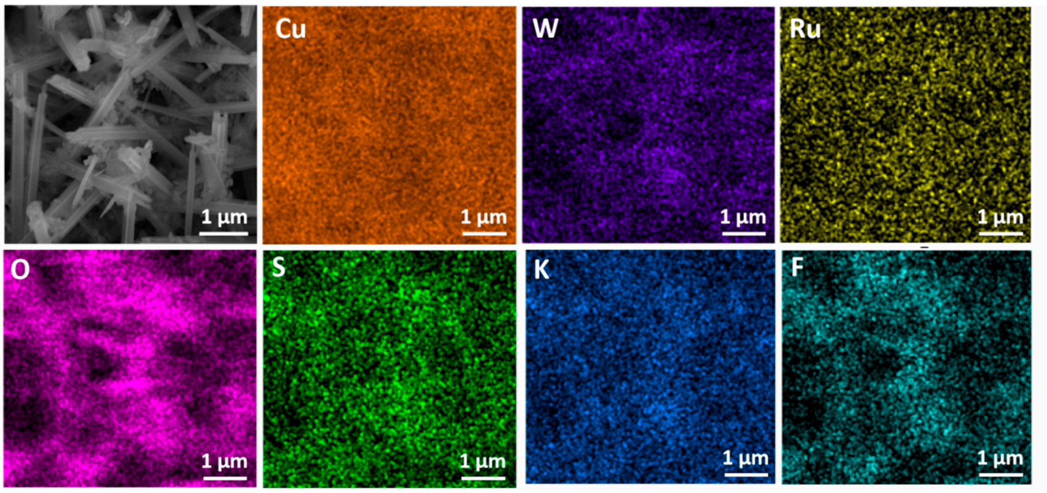

Figure S16. SEM and EDS elemental mapping of the Ru/WO_3/$Cu(OH)_2$/FC electrode after long-term chronoamperometry tests.

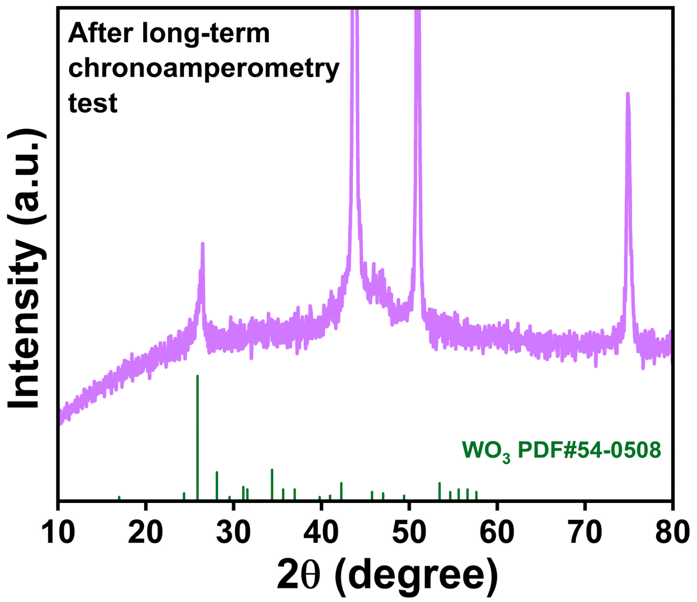

Figure S17. XRD pattern of the Ru/WO3/Cu(OH)2/FC electrode after long-term chronoamperometry tests.

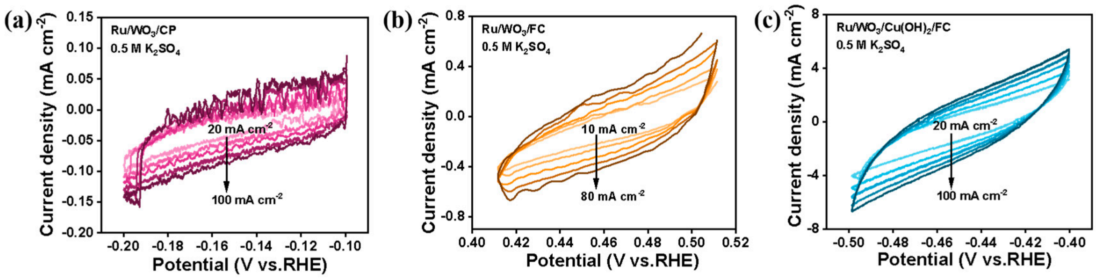

Figure S18. CV curves of (a) Ru/WO3/CP, (b) Ru/WO3/FC, (c) Ru/WO3/Cu(OH)2/FC at different scan rate.

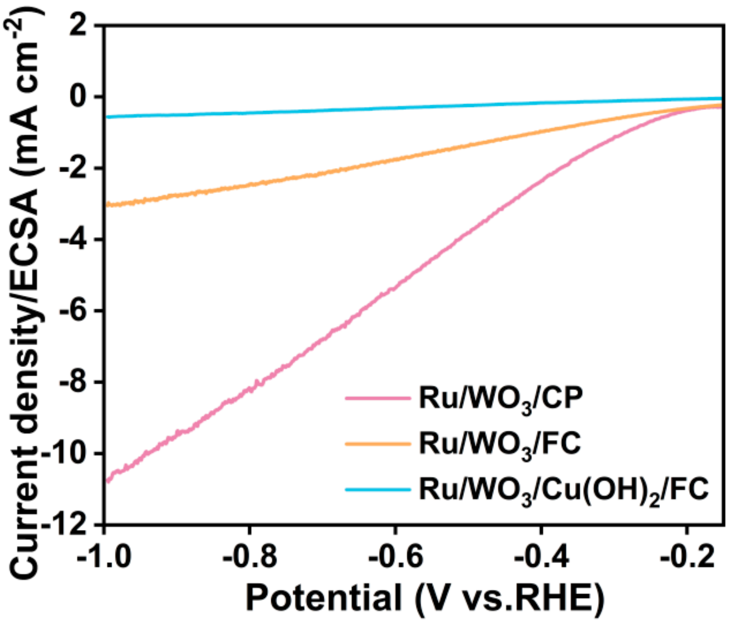

Figure S19. LSV curves of $Ru/WO_3/CP$, $Ru/WO_3/FC$, and $Ru/WO_3/Cu(OH)_2/FC$ in a 0.5 M K_2SO_4 + 50 mM KNO_3 electrolyte, normalized according to ECSA.

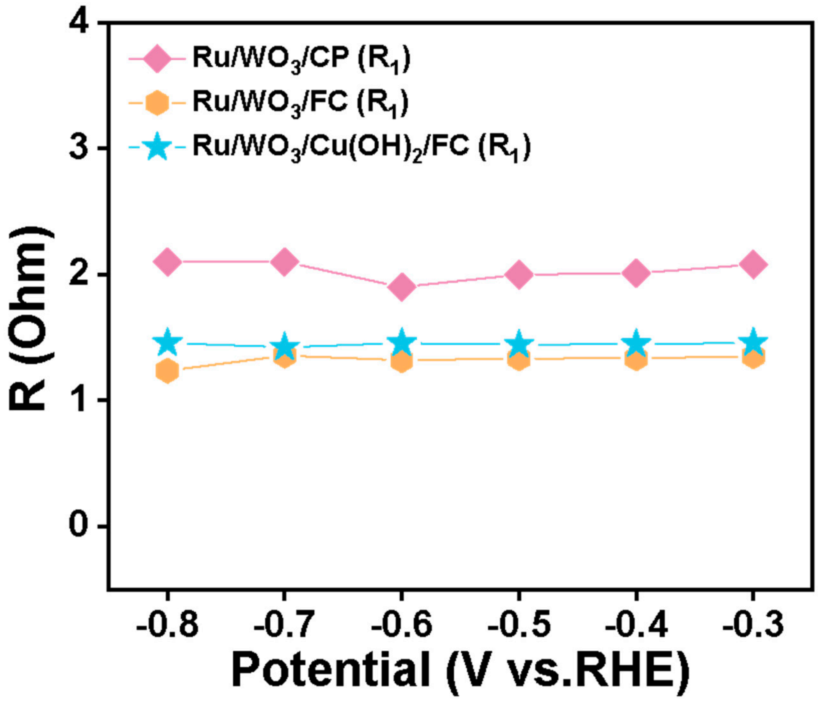

Figure S20. Ohmage of $Ru/WO_3/CP$, $Ru/WO_3/FC$ and $Ru/WO_3/Cu(OH)_2/FC$ in 0.5 M K_2SO_4 + 50 mM KNO_3 at OCP.

Table S1. Statistical analysis of NO_3RR performance for different catalysts in neutral electrolytes

Catalyst	Electrolyte	Potential (V vs.RHE)	NH_3 yield rate (mg h^{-1} cm^{-2})	FE (%)	Ref.
**$Ru/WO_3/Cu(OH)_2$/FC**	**0.5 M K_2SO_4 + 0.05 M KNO_3**	**-0.8**	**16.1**	**75.8**	**This work**
Ru/MSN-WO_{3-x}	0.1 M KNO_3 + 0.25 M K_2SO_4 + 0.5 M phosphate	-0.6	12.4	80.0	1
CuCo-in-CNT	0.1 M Na_2SO_4 + 100 mg/L NO_3^-–N	−0.7	2.2	93.8	2
1D $MnFeO_x$	K_2SO_4 +0.05 M NO_3^-	-1.0	12.3	95.9	3
FeNC-Fe_3O_4	0.25 M Na_2SO_4 + 0.1 M KNO_3	-0.6	3.0	95.6	4
R-TiO_2/Cu_2O	1 M Na_2SO_4 +50 ppm NO_3^-–N	-0.5	3.9	85.7	5
Qc-5-CuCo	0.3 M Na_2SO_4 + 0.1 M $NaNO_3$	0.6	6.0	96.4	6
Co–CuAl LDHs	0.1 M KPi + 50 mM KNO_3	-0.7	1.3	80.7	7
Co-CNFs@Ag	0.5 M K_2SO_4 + 0.05 M KNO_3	-0.89	4.5	58.1	8
Cu@bpy-COF	0.5 M K_2SO_4 + 0.1 M KNO_3	-0.85	17.4	93.6	9
Mo−C_1N_2	0.1 M KNO_3 + 0.1 M K_2SO_4	-0.8	12.0	96.3	10

References

1. Fu H., Lu S, Xin Y., Xiao S. Xiao, Chen L., Li Y., Shen K, In situ bulk hydrogen intercalation in a mirrorsymmetric Ru/WO_{3-x} nanoarray boosts neutral electrocatalytic nitrate reduction to ammonia, *Energy Environ. Sci.*, **2025**, *18*, 818-830.
2. Meng L., Shen C., Zhou M., Wang X., Liu Y., Wang C., Liu M., Quan X., Liu Y., Steering the nitrate electroreduction pathway via nanoconfinement-induced hydrogen-bond network regulation, *Nat. Water*, **2026**, *4(4)*, 481-492.
3. Jia X., Kong Y., Wan D., Liu L., He S., Liu X., Yang H., Hu Q., Zhang X., He C., Self-Sustaining Dynamic Alkaline Microenvironment-Mediated Efficient Nitrate Electroreduction to Ammonia on MnFeOx in Neutral Electrolyte, *Angew. Chem. Int. Ed.* **2026**, e4598330.
4. Wei J., Lu X., Lin H., Liu S., Wei X., Sun M., Liu G., Hu C., Wang L., Zhao H., Li Y., Coupling iron oxide with iron single-atom catalyst promotes neutral electrochemical nitrate reduction from water, *Water Research*, **2025**, 124936.
5. Wang J., Dong W., Song Q., Zhang S., Li M., Chen J., Zhang S., Lu J., Unveiling heterointerface activation effects with different titanium dioxide crystal phases for electrocatalytic nitrate-to-ammonia reduction, *J. Hazard. Mater.*, **2025**, *488*, 137174.
6. Wang M., Li W., Qiao H., Yu Y., Ning F., Liu Y., Qiao J., Qc-5-Cu_xCo_y MOFs for boosting electrochemical nitrate reduction to ammonia in neutral electrolyte, *Process Saf. Environ.*, **2026**, 109110.
7. Sim G., Park J., Byun J. S., Seok J. H., Kim Y., Park J., Liu R., Ng Y. H., Oh H., Yoo C., Kim J. K., Lee S. U., Yang W., Atomic scale distortion enables optimal Cu-Co coupling for selective electrochemical nitrate to ammonia conversion in neutral electrolytes, *Nano Energy*, **2026**, 112035.
8. Wang D., Qi R., Deng L., Fan C., Huang L., Chen Z., Lu X., Dual metal synergy promotes electrocatalytic nitrate-to-ammonia conversion in neutral electrolyte. *J. Colloid Int. Sci.*, **2026**: 140536.
9. Zhao K., Sun Y., Yang X., Pu J., Shi Y., Chen P., Yang J., Yu F., Unsaturated Cu single-atom catalysts in COFs for efficient neutral ammonia electrosynthesis from nitrate. *ACS Sustainable Chem. Eng.*, **2026**, *14(7)*: 3342-3353.
10. Liang S., Bui T. S., Ma Z., Han C., Zhou S., Webster R. F., Shan Y., Liu J., Zhang Q., Lu X., Lovell E., Asymmetric coordination in Mo single-atom catalysts drives selective electrochemical nitrate reduction to ammonia. *Appl. Catal. B: Environ.*, **2026**: 126962.
